# A Novel Approach to Pharmacodynamic Assessment of Antimicrobial Agents: New Insights to Dosing Regimen Design

**DOI:** 10.1371/journal.pcbi.1001043

**Published:** 2011-01-06

**Authors:** Vincent H. Tam, Michael Nikolaou

**Affiliations:** 1 Department of Clinical Sciences and Administration, College of Pharmacy, University of Houston, Houston, Texas, United States of America; 2 Department of Chemical and Biomolecular Engineering, Cullen College of Engineering, University of Houston, Houston, Texas, United States of America; University of California San Diego, United States of America

## Abstract

Pharmacodynamic modeling has been increasingly used as a decision support tool to guide dosing regimen selection, both in the drug development and clinical settings. Killing by antimicrobial agents has been traditionally classified categorically as concentration-dependent (which would favor less fractionating regimens) or time-dependent (for which more frequent dosing is preferred). While intuitive and useful to explain empiric data, a more informative approach is necessary to provide a robust assessment of pharmacodynamic profiles in situations other than the extremes of the spectrum (e.g., agents which exhibit partial concentration-dependent killing). A quantitative approach to describe the interaction of an antimicrobial agent and a pathogen is proposed to fill this unmet need. A hypothetic antimicrobial agent with linear pharmacokinetics is used for illustrative purposes. A non-linear functional form (sigmoid Emax) of killing consisted of 3 parameters is used. Using different parameter values in conjunction with the relative growth rate of the pathogen and antimicrobial agent concentration ranges, various conventional pharmacodynamic surrogate indices (e.g., AUC/MIC, Cmax/MIC, %T>MIC) could be satisfactorily linked to outcomes. In addition, the dosing intensity represented by the average kill rate of a dosing regimen can be derived, which could be used for quantitative comparison. The relevance of our approach is further supported by experimental data from our previous investigations using a variety of gram-negative bacteria and antimicrobial agents (moxifloxacin, levofloxacin, gentamicin, amikacin and meropenem). The pharmacodynamic profiles of a wide range of antimicrobial agents can be assessed by a more flexible computational tool to support dosing selection.

## Author Summary

Antimicrobial agents have been the mainstay of treatment for a variety of infectious diseases such as urinary tract infections and pneumonia. Due to the increasing incidence of antimicrobial resistance, there is an ever demanding need to develop new antimicrobial agents rapidly. These agents can be given in different ways, both in terms of the daily dose and dosing frequency. The traditional approach to the design of antimicrobial agent dosing regimen relies primarily on a categorical classification, which often could be restrictive. We proposed a new computational method to provide quantitative insights to the interaction between an antimicrobial agent and a pathogen (pharmacodynamics). With a more robust understanding of this relationship, the effectiveness of different antimicrobial dosing regimens can be compared efficiently, which would facilitate new agent development by rationally guiding dosing regimen selection. The relevance of our approach was supported by a series of experimental validation using different antimicrobial agents and bacteria. A higher probability of resistance suppression could be achieved with optimal dosing regimens, which may prolong the clinical utility of new agents under development.

## Introduction

Microbial resistance is rising at an alarming rate, rendering many antimicrobial agents ineffective. There is an ever demanding need to develop new antimicrobial agents and optimize available agents to curb the rising resistance prevalence. There are experimental and clinical evidence that dosing exposure could have an impact on patient outcomes and the development of resistance [1], [2]. As a result, pharmacodynamic modeling has been increasingly used as a decision support tool to guide dosing selection in new drug development [3].

Microbial killing by unbound antimicrobial agents has been traditionally classified categorically as concentration-dependent or time-dependent. The pharmacodynamics of many antimicrobial agents are well accepted to be linked to surrogate indices such as peak concentration (Cmax)/minimum inhibitory concentration (MIC), area under the concentration-time profile (AUC)/MIC or the proportion of dosing interval in which the concentration is above the MIC (%T>MIC) [4]. Dosing regimen design could be based on results from traditional dose fractionation studies [5]–[7]. As long as the pharmacokinetics of the antimicrobial agent is linear, the entire daily dose could be given once daily, half the daily dose given twice daily, one-third the daily dose given three times each day, etc. to achieve a similar daily AUC. Depending on the difference in outcomes observed (if any), different categorical pharmacodynamic characterization could be deduced. For instance, if the once-daily regimen is the most effective, Cmax/MIC would be most likely linked to outcomes. If the most frequent dosing regimen is found to be the most beneficial, %T>MIC [or minimum concentration (Cmin)/MIC] would likely be the most useful pharmacodynamic surrogate index predicting outcomes. If all the dosing regimens are similar, AUC/MIC would be deemed to be associated with outcomes. This traditional approach has been applied in a wide range of infection types, patient groups and duration of therapy [1]; heterogeneous bacterial populations at the infection site have also been considered [8].

While the dose fractionation design is intuitive and commonly used, there may be situations where such a categorical approach to pharmacodynamic assessment is overly restrictive. It is especially so with new drugs (or drug classes) with complex pharmacokinetics and pharmacodynamics. We propose an alternative approach to pharmacodynamic characterization of the interaction between an antimicrobial agent and a pathogen. Instead of relying on time-dependent endpoints such as Cmax or MIC, the time course of a bacterial population when subjected to an antimicrobial agent exposure is captured by a dynamic mathematical model. The advantages of such a modeling approach have been reviewed previously [3], [9]. The proposed pharmacodynamic characterization can be subsequently linked to a novel computational method to derive the dosing intensity of a dosing regimen, which would facilitate objective comparison of various dosing regimens [10]. To illustrate our approach in the clearest way possible, a hypothetic drug is used via a series carefully constructed computer simulations. The relevance of our approach is subsequently supported by application to several experimental datasets we have reported in the past.

## Methods

### Basic assumptions

**Figure 1:**
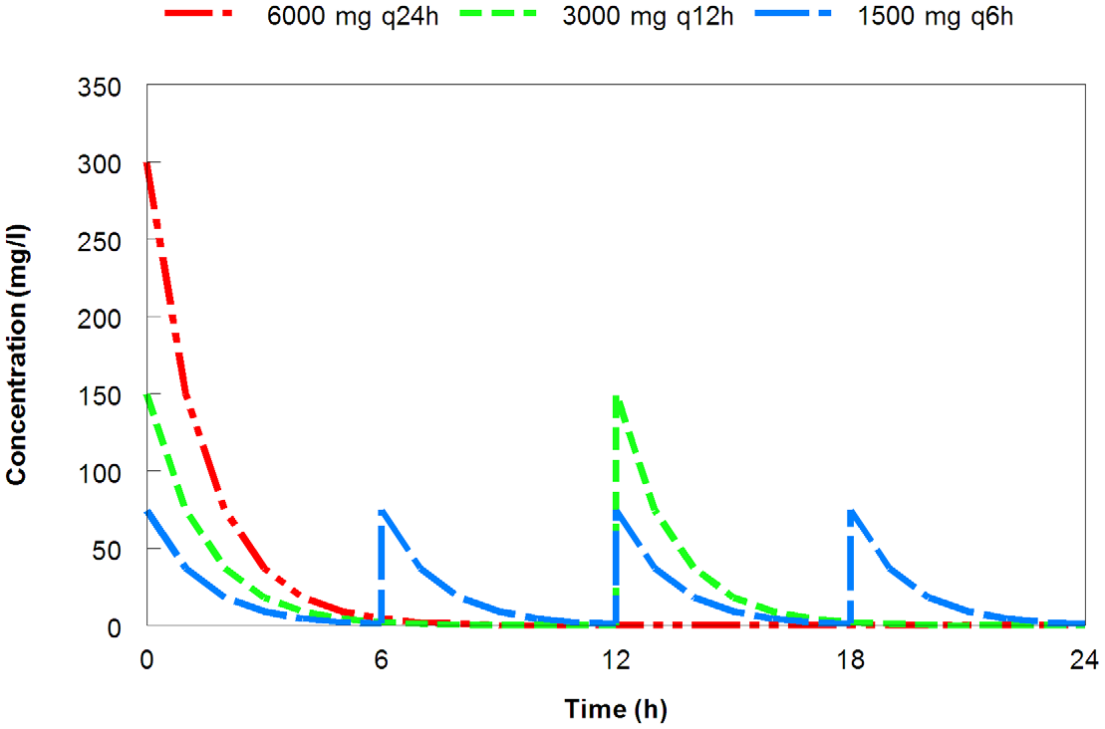
Dose fractionation designs of an identical daily dose.

A hypothetic drug is used for illustrative purposes. The pharmacokinetics of this drug is linear and characterized by a one-compartment intravenous bolus model. The volume of distribution of this drug is 20 liters, with an elimination half-life of 1 hour and negligible protein binding. A daily dose of 6000 mg can be given once daily (6000 mg q24h), twice daily (3000 mg q12h) or 4 times daily (1500 mg q6h). The respective serum concentration-time profiles and exposures achieved are as shown in Figure 1 and Table 1. In addition, the target pathogen is assumed to have a growth rate (*K_g_*) of 1.0 h^−1^ (microbial doubling time of approximately 42 minutes); resistance is not expected to incur a significant biofitness cost (no change in bacterial growth rate over time).

**Table 1:** Dose fractionation designs of an identical daily dose.

Dosing regimen	Cmax (mg/l)	AUC_24_ (mg.h/l)	% T>4mg/l
6000 mg q24h	300	433	25
3000 mg q12h	150	433	42
1500 mg q6h	75	433	67

Note: AUC_24_ may not be identical in drugs with an elimination half-life of ≥4 hours. The discrepancy is due residual drug at the end of the 24 hour period, which is more prominent with a drug of long half-life. One simple way to circumvent the discrepancy is to focus on AUC _0–infinity_ (total cumulative exposure) instead of AUC _0–24_.

### General experimental design

Details of the experimental setup have been reported previously [11]–[13]. Briefly, a heterogeneous bacterial inoculum (consists of multiple sub-populations associated with different susceptibility - a function of the total bacterial burden and the mutational frequency to resistance of the isolate being studied) is exposed to different and escalating drug concentrations; the drug concentration in each experiment is constant over time. Serial samples are taken from various flasks over 24 hours to determine viable bacterial burden by quantitative cultures. A dynamic mathematical model is fit to the data to derive the best-fit model parameter estimates. The growth rate of the target pathogen can be derived from placebo control experiments. Regrowth observed after an initial decline in bacterial burden is attributed to adaptation of the bacterial population under a selective pressure (i.e., enrichment of sub-populations with reduced susceptibility over time, resulting in resistance emergence during therapy). It should be stressed that the term ‘adaptation’ is used here to denote adaptation at the population level, not at the individual cell level (e.g., through induction of efflux pumps or beta-lactamases). Namely, the entire population adapts because of a selective pressure. Various specific mathematical structures have been used, an example is shown in one of our previous work [12]. Using the pharmacodynamic profile of the most resistant cell in the population (i.e., the killing function when *t*→∞), assessments are made as to which of the proposed dosing regimens (detailed above) would have the greatest bactericidal activity. A dosing regimen with a greater average kill rate is expected to have a higher probability of suppressing the development of resistance over time, when the majority of the population can be controlled. Subsequently, the same inoculum of bacteria is exposed to various fluctuating drug concentration-time profiles in a hollow-fiber infection model. Experimentally observed bacterial responses are used to support the validity the categorical predictions of the mathematical model.

### Mathematical modeling

The saturable killing rate of an antimicrobial agent is characterized by a sigmoid Emax model, commonly used in many investigations [8], [14]–[18]. A new computational approach is proposed to facilitate objective comparison. The average kill rate of a dosing regimen can be conceptualized as the dosing intensity. It is derived by converting instantaneous drug concentration to instantaneous kill rate using the parameter estimates of the kill function, and subsequently integrating all instantaneous kill rates with respect to time over a dosing interval [19].
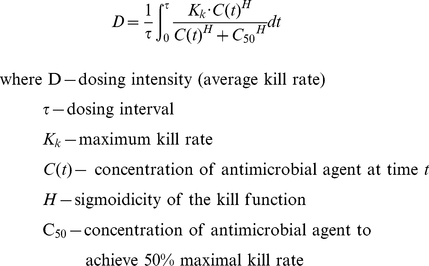



The average kill rate can be used quantitatively to compare the effectiveness of various dosing regimens, in relation to the growth rate of the target pathogen. This approach assumes eradication of the bacterial population cannot be achieved within one dosing interval; a large killing rate shortly after dose administration is as important as a large killing rate towards the end of the dosing interval. The importance of the average kill rate (*D*) is that complete eradication of the entire bacterial population and prevention of resistance emergence will be observed, if *D>K_g_* for the most resistant cell in the population. The preceding statement assumes that no physiological changes (e.g., induction of efflux pumps or beta-lactamases) take place in bacteria as a result of exposure to the antimicrobial agent. All simulations were performed using the ADAPT II program [20], and graphs were plotted with Mathematica 6.0 (Wolfram Research, Inc., Champaign, IL).

## Results

### Pharmacodynamic profiles

**Figure 2:**
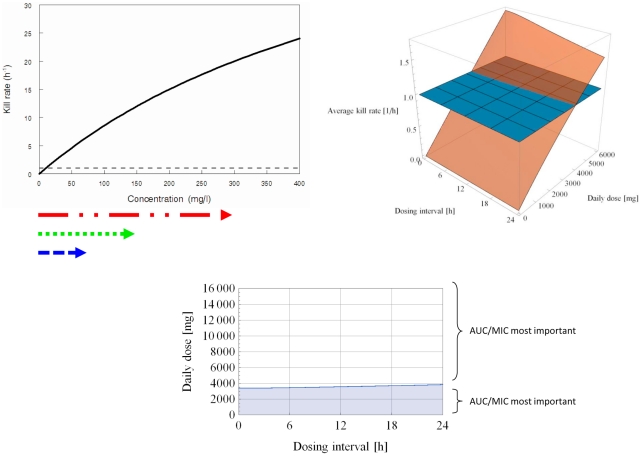
Concentration dependent killing; AUC/MIC most important. *K_k_* = 60.0 h^−1^. *C_50_* = 600.0 mg/l. *H* = 1.0. Black solid line depicts the relationship between drug concentration and killing rate; black dotted line represents the microbial growth rate. Arrows below represent concentration ranges achieved with various dosing regimens (red – once daily; green – twice daily; blue – four times daily). Two intersecting planes are shown: a translucent surface and an opaque mesh surface (where the average kill rate = 1.0 h^−1^). The 3-dimensional mesh surface is made up of a collection of data points; each datum point is characterized by a value on the x, y and z axes, corresponding to the daily dose (x), dosing interval (y) and average kill rate (z). For a dosing regimen to suppress resistance development, it is imperative that the average kill rate (*D*) is more than the growth rate (*K_g_*) of the target pathogen. To identify promising dosing regimens (combinations of dose and dosing interval) to suppress resistance development, *D* must be greater than *K_g_* (the region where the translucent surface is above the opaque mesh plane). White area depicts dosing regimens (combinations of daily dose and dosing interval which the average kill rate is >1.0 h^−1^. Using a daily dose of 6000 mg, the average kill rates for different regimens are: 1.463 h^−1^ (q24h), 1.610 h^−1^ (q12h), and 1.690 h^−1^(q6h).

Using the same structural form, three typical pharmacodynamic profiles can be shown (Figures 2–4). These distinct pharmacodynamic profiles are characterized by 3 parameter estimates and represent unique (extreme) situations. In reality, intermediate profiles (e.g., partially concentration dependent killing) are also possible and could be objectively described by these parameter estimates. The growth rate of the target pathogen is shown concurrently for comparison. Finally, the concentration ranges of antimicrobial agent attained by different dosing regimens are also shown in each situation.

**Figure 3 pcbi-1001043-g003:**
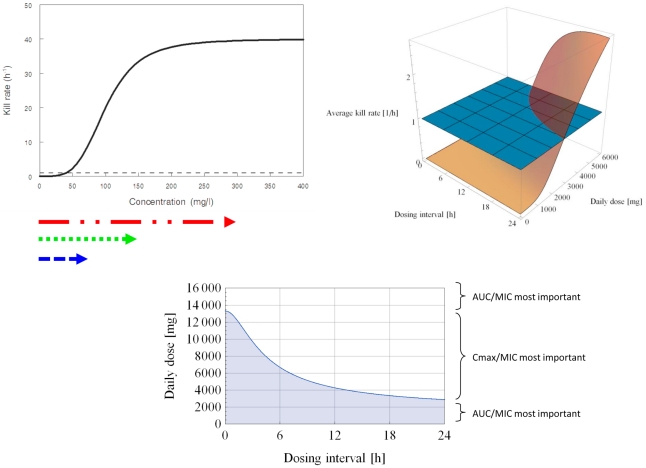
Concentration dependent killing; Cmax/MIC most important. *K_k_* = 40.0 h^−1^. *C_50_* = 100.0 mg/l. *H* = 4.0. Black solid line depicts the relationship between drug concentration and killing rate; black dotted line represents the microbial growth rate. Arrows below represent concentration ranges achieved with various dosing regimens (red – once daily; green – twice daily; blue – four times daily). White area depicts dosing regimens (combinations of daily dose and dosing interval which the average kill rate is >1.0 h^−1^. Using a daily dose of 6000 mg, the average kill rates for different regimens are: 2.650 h^−1^ (q24h), 2.168 h^−1^ (q12h), and 0.689 h^−1^(q6h). Using a conventional dose fractionation design with 16000 mg daily (e.g., 16000 mg q24h, 8000 mg q12h, 4000 mg q6h, etc.), all regimens are expected to suppress the bacterial population, thus AUC/MIC is likely to be concluded as the pharmacodynamic index associated with resistance suppression. In addition, if a daily dose of 2000 mg is selected (e.g., 2000 mg q24h, 1000 mg q12h, 500 mg q6h, etc.), all regimens are expected to be associated with regrowth, and therefore AUC/MIC is also likely to be deemed as the pharmacodynamic index associated with resistance development. However, if a daily dose of 6000 mg is chosen (e.g., 6000 mg q24h, 3000 mg q12h, 1500 mg q6h, etc.), a less frequent dosing regimen (e.g., q24h) is anticipated to have a higher likelihood of suppressing resistance, and as such Cmax/MIC is likely to be concluded as the pharmacodynamic index associated with resistance suppression. Therefore, the strict use of surrogate indices in pharmacodynamic modeling is not always optimal as they may be subjected to selection basis of the concentration range examined.

**Figure 4:**
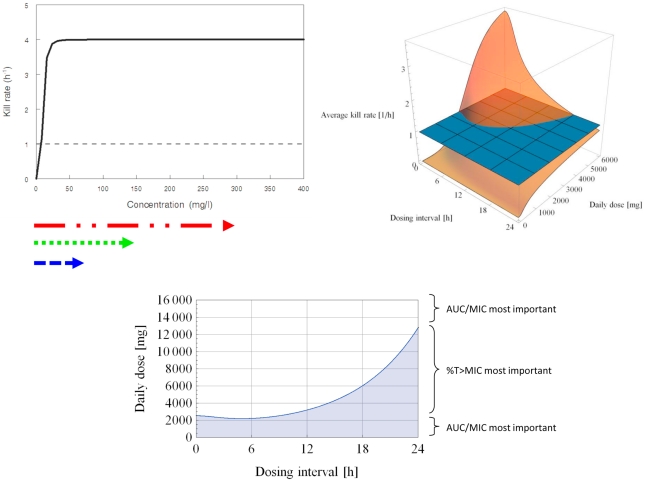
Time dependent killing; %T>MIC most important. *K_k_* = 4.0 h^−1^. *C_50_* = 10.0 mg/l. *H* = 4.0. Black solid line depicts the relationship between drug concentration and killing rate; black dotted line represents the microbial growth rate. Arrows below represent concentration ranges achieved with various dosing regimens (red – once daily; green – twice daily; blue – four times daily). White area depicts dosing regimens (combinations of daily dose and dosing interval which the average kill rate is >1.0 h^−1^. Using a daily dose of 6000 mg, the average kill rates for different regimens are: 0.818 h^−1^ (q24h), 1.303 h^−1^ (q12h), and 1.950 h^−1^(q6h).

In Figure 2, the maximal killing rate is significantly higher than the growth rate of the target pathogen. The concentration-effect relationship is also fairly linear in the concentration ranges achieved by all the dosing regimens. Therefore, attaining a high concentration for a brief period of time would not be much more advantageous, as compared to lower concentrations for a more prolonged time frame. As a result, dosing frequency is not expected to have a huge impact of the overall bactericidal activity, as long as the daily dose is kept constant. Using conventional nomenclature, this pharmacodynamic profile is concentration dependent killing, and AUC/MIC would be considered the most important. This pattern is consistent with our experience with data reported for moxifloxacin [21] and levofloxacin against *Escherichia coli* (unpublished data).

In Figure 3, the maximal killing rate is still significantly higher than the growth rate of the target pathogen. However, the concentration-effect relationship is non-linear in the concentration range achieved by the dosing regimens. The killing rate rises sharply beyond a certain threshold (i.e., approximately 75 mg/l - the peak concentration of the q6h dosing regimen). A dosing regimen attaining a concentration beyond this threshold could encounter a more than proportional increase in killing rate, even for a brief period of time. Consequently, less frequent dosing (to achieve a concentration transiently above the threshold) is expected to result in a greater overall bactericidal activity. Using conventional nomenclature, this pharmacodynamic profile is concentration dependent killing, and Cmax/MIC would be considered the most important.

An important point should be raised here. The above example is illustrated with only one daily dose (6000 mg daily). Using a qualitative (yes / no) outcome assessment (e.g., mortality, clinical cure, resistance suppression), different categorical interpretations may be arrived at with the same pharmacodynamic profile. To further exemplify this point, the average kill rate is graphed as a function of both daily dose and dosing interval, using a 3-dimensional surface response as detailed previously [22]. In addition, a cross sectional view is undertaken when the average kill rate equals to the growth rate of the pathogen (*D* = *K_g_* = 1.0 h^−1^). When the average kill rate of a dosing regimen is greater than the growth rate of the target pathogen (i.e., *D*>*K_g_*), resistance suppression is generally anticipated. On the other hand, if the average kill rate of a dosing regimen is less than the growth rate of the target pathogen (i.e., *D*<*K_g_*), resistance amplification is expected over time. Given that AUC and Cmax are highly correlated in clinical or animal studies (where drug clearance cannot be easily modified), it may be difficult to discriminate whether AUC/MIC or Cmax/MIC is the pharmacodynamic variable most closely linked to outcomes. Our analysis revealed that there could be experimental evidence supporting both conclusions in the same antimicrobial agent-pathogen combination. The daily dose(s) to be examined in fractionation studies should also be carefully considered in the analysis. The dose exposure used may not be the same for different outcomes (e.g., survival, 1-log drop in microbial burden, or resistance suppression), and thus may have contributed (at least partially) to enthusiastic debates in the literature [23]–[26]. Other investigators have also reported different pharmacodynamic characterization at different dose levels of the same drug. Fractionation of the minocycline dose at the human dose equivalents showed no difference between once, twice, or three times a day dosing against *Staphylococcus aureus* (i.e., AUC/MIC was the most important). In contrast, fractionation of the dose with a static effect indicated that once daily dosing was superior (i.e., Cmax/MIC was the most important) [27]. In addition, these conflicting patterns are also consistent with our own experience examining the pharmacodynamics of the aminoglycosides. Using a dose fractionation design, there were data to support gentamicin AUC/MIC was the most important against *Pseudomonas aeruginosa* [11]; but amikacin Cmax/MIC appeared to be the most important against *Acinetobacter baumannii* [13].

Finally in Figure 4, the maximal killing rate is only slightly higher than the growth rate of the target pathogen. The concentration-effect relationship is non-linear in the concentration range achieved by the dosing regimens, but the threshold which the killing rate rises sharply (i.e., approximately 5 mg/l) could be readily attained by all 3 dosing regimens. Under such circumstances, a transiently high concentration achieved in a less frequent dosing regimen does not translate to a higher kill rate, and killing is compromised when the concentration falls below the threshold for a prolonged period of time. Consequently, a more rational dosing strategy is to maintain concentrations above the threshold for as long as possible. Using conventional nomenclature, this pharmacodynamic profile is time dependent killing, and %T>MIC (or Cmin/MIC) would be considered the most important when the investigations are performed in the ‘critical’ daily dose range. In the extremes of the daily dose range (i.e., more than 14000 mg or less than 2000 mg daily), AUC/MIC could still be interpreted as an important surrogate index linked to outcomes observed. This pattern is consistent with our experience with data reported for meropenem against *Pseudomonas aeruginosa* [28].

## Discussion

In all 3 typical pharmacodynamic profiles described, the same structural form of killing rate was used (a sigmoid Emax model). Different profiles were simply reflected in the values of the model parameters, which could be derived from actual experimental data observed between an antimicrobial agent and a pathogen within a short timeframe (e.g., 24 hours). Of note, knowledge of specific mechanism(s) of resistance was not necessary as inputs. As shown above, an antimicrobial agent can be shown to exhibit both concentration and time-dependent killing, but the proposed model was flexible enough to describe different distinct pharmacodynamic profiles (and any intermediates in between).

Antimicrobial kill kinetics has been previously examined using a related approach. In one study, concentration dependency of bacterial killing was primarily attributed to the sigmoidicity constant (i.e., *H* in our model) [29]. Drugs exhibiting concentration-dependent killing was associated with a low sigmoidicity constant. In our opinion, all drugs exhibit concentration-dependent killing in the concentration range corresponding to 20%–80% of maximal killing rate (regardless of the magnitude of sigmoidicity). It is equally important to consider *K_k_* and *C_50_*, in order to get a complete and accurate pharmacodynamic assessment of an antimicrobial agent. As reviewed previously by Czock *et al* [30], the effect of the sigmoidicity constant could be influenced by the ratio of *K_k_*/*K_g_* (similar illustrations were also shown in Figures 3 and 4). In addition, whether such concentration-dependent killing is clinically relevant is further dependent on whether the concentration-dependent killing concentration range is achievable in humans with acceptable toxicity, potentially resulting in other categorical descriptions on a continuous spectrum such as “partially concentration-dependent” (where there is partial overlap of the concentration ranges) or “time-dependent” killing (where there is minimal overlap of the concentration ranges). Thus *C_50_* is also not irrelevant as pointed out previously [30]. Consequently, we resorted to a more comprehensible approach by taking into consideration additional pertinent variables. We attempted to develop a more robust computational tool covering a wider spectrum of relevant scenarios in new drug development.

To extend these pharmacodynamic concepts previously reviewed, we put forth herein a novel concept to derive the dosing intensity of a dosing regimen by comparing the average killing rate to the growth rate of the target pathogen. A similar approach was proposed in linking pharmacokinetics to drug effects in circular / proliferative systems using the concept of reproduction minimum inhibitory concentration (RMIC) and equivalent effective constant concentration (ECC) [31]. As we have shown in Figure 2, in the unique case of linear concentration-effect relationship, the heuristics proposed previously (RMIC and ECC) would be identical. Dosing frequency is not expected to have a major impact of effectiveness, as long as the daily dose is kept constant (i.e., AUC/MIC is the most important). To highlight a common drawback in conventional modeling, our computational tool illustrated the pharmacokinetic / pharmacodynamic concepts via several theoretical and experimental case examples.

We believe our proposed approach would enhance the applicability of these concepts in drug development and clinical settings. Specifically, the pharmacodynamics of antimicrobial agents are characterized as a continuum (as opposed to discrete categories), allowing relatively simple numeric computational methods to be applied. The efficiency to objectively compare the effectiveness of a large number of dosing regimens (to be investigated in pre-clinical or clinical studies) would be improved as a result. Furthermore, we have also justified our rationale using supportive data derived under more clinically relevant experimental conditions (multiple sets of experimental data involving fluctuating drug concentrations over at least 72 hours, as shown in Table 2). Such an approach provides a fresh perspective on our understanding of antimicrobial agent pharmacodynamics; the new insights could resolve heated debates in the literature relating to which pharmacodynamic surrogate index is the most closely related to outcomes.

**Table 2:** Summary of selected dose fractionation studies to suppress bacterial resistance development.

Bacteria	*E. coli*	*P. aeruginosa*	*A. baumannii*	*P. aeruginosa*
Drug	Levofloxacin	Gentamicin	Amikacin	Meropenem
Design	Similar AUC_24_/MIC (45 and 10); q24h vs q12h	Similar AUC_24_/MIC (40); q24h vs q8h	Similar AUC_24_/MIC (72); q24h vs q8h	Similar Cmax/MIC (64) q8h; Cmin/MIC 6 vs 2
Results	q24h similar to q12h at both AUC_24_/MIC; AUC_24_/MIC 10 failed to suppress resistance development; AUC_24_/MIC 45 suppressed resistance development	q24h similar to q8h; both regimens failed to suppress resistance development	q24h superior to q8h in suppressing resistance development	Cmin/MIC 6 superior to 2 in suppressing resistance development
Categorical interpretation	AUC/MIC most important	AUC/MIC most important	Cmax/MIC most important	AUC/MIC or Cmin/MIC most important
Reference	Unpublished data	[11]	[13]	[28]

In this study, the mathematical model used was to characterize the behavior of a heterogeneous bacterial inoculum, which consists of multiple sub-populations associated with different susceptibility. This modeling approach (involving a time-variant parameter to account for adaptation of the bacterial population) would enable us to better capture regrowth and / or emergence of resistance over time. Nonetheless, the mathematical model is flexible and can be modified easily to accommodate a homogeneous inoculum by not allowing *C_50_* (an index of bacterial population susceptibility) to increase over time (no adaptation). Regardless of the approach used, fundamental limitations in dose fractionation design and categorical pharmacodynamic classification of antimicrobial agents would still remain. However, the model does not account for the immune system (which may play an important role against small resistant populations) and bacterial stress response not observed during the initial observation period. Both issues (modeling the effect of the immune system and temporal bacterial response leading to resistance) are currently under investigation.

In summary, pharmacodynamic modeling is an important decision-support tool to guide the selection of dosing regimens. The use of surrogate pharmacodynamic indices has taught us much on the differences in the killing profiles of different antimicrobial agents, resulting in several rational dosing strategies to optimize patient outcomes. As we are dealing with drugs with more complex pharmacokinetics and pharmacodynamics, it is also clear that using simple surrogate pharmacodynamic indices may not always be informative enough to make good decisions to dosing selection. In the interest of further accelerating new drug development and for the benefits of our patients, alternative modeling and computational approaches, such as the one proposed herein should be explored.
